# How Does Ambient Light Affect the Image Quality of Phosphor Plate Digital Radiography? A Quantitative Analysis Using Contemporary Digital Radiographic Systems

**DOI:** 10.3390/s22228627

**Published:** 2022-11-09

**Authors:** Matheus Sampaio-Oliveira, Luiz Eduardo Marinho-Vieira, Victor Aquino Wanderley, Gláucia Maria Bovi Ambrosano, Ruben Pauwels, Matheus L. Oliveira

**Affiliations:** 1 Department of Oral Diagnosis, Division of Oral Radiology, Piracicaba Dental School, University of Campinas (UNICAMP), Piracicaba 13414-903, Brazil; 2 Department of Community Dentistry, Division of Biostatistics, Piracicaba Dental School, University of Campinas (UNICAMP), Piracicaba 13414-903, Brazil; 3 Aarhus Institute of Advanced Studies, Aarhus University, 8000 Aarhus, Denmark; 4 Department of Radiology, Faculty of Dentistry, Chulalongkorn University, Bangkok 10330, Thailand

**Keywords:** digital dental radiography, light, image processing

## Abstract

The aim of this study is to quantitatively evaluate the influence of the duration of ambient light exposure on the image quality of digital radiographs obtained with contemporary phosphor plate (PSP)-based systems. Radiographs of an aluminum step-wedge were obtained using VistaScan and Express systems at five X-ray exposure times: 0.10, 0.20, 0.32, 0.40, and 0.50 s; the resulting dose-area products were, respectively, 21.93, 43.87, 70.19, 87.75, and 109.6 mGycm^2^. Before PSP read-out, half of the sensitive surface of the PSP plates was exposed to ambient light for 5, 10, 30, 60, and 90 s. The effect of light exposure on brightness, contrast, contrast-to-noise ratio (CNR), signal-to-noise ratio (SNR), and image saturation was compared using ANOVA with the Tukey test (α = 0.05). Ambient light exposure increased brightness and contrast and reduced CNR and SNR in PSP-based radiographs of contemporary digital systems. At the longest X-ray exposure times, ambient light exposure reduced the dark saturation (mean gray values ≤ 1) observed in Express. In conclusion, the negative effects of ambient light observed on the image quality of PSP-based radiographs are directly proportional to the duration of exposure. Clinicians should be aware of such harmful effects when handling and scanning PSP plates in bright environments.

## 1. Introduction

Photostimulable phosphor (PSP) plate is a type of image receptor widely used in intraoral digital radiography. After exposure to X-rays, the latent image stored in the PSP plate needs to be scanned to produce a visible image on the computer screen [1,2]. Despite the benefits of PSP systems, such as time-saving, post-processing tools, and good image quality [3], this inherently indirect way of producing radiographs may be a potential source of image-quality impairment. Because PSP plates are moderately sensitive to light, ambient lighting can lead to fading, density inhomogeneity, and noise [4,5,6,7,8].

Fading can be described as the natural loss of the stored energy from within a phosphor crystal lattice. In PSP plates, fading of the trapped signal occurs exponentially over time as a result of spontaneous phosphorescence, which is the transformation of Eu^3+^ to Eu^2+^ [9,10]. This effect can occur when the scanning is delayed and/or when the PSP plate is exposed to ambient light before scanning [2,11]. Previous studies assessing the impact of fading on the image quality of radiographs obtained using PSP-based systems have been published [2,9,11,12,13]; most of them [9,11,12,13] focused only on the effect of delayed scanning on mean gray values (MGVs). Conversely, in 2004, Ramamurthy et al. [2] evaluated the impact of ambient light exposure on PSP plates of two discontinued digital radiographic systems but considered only the signal-to-noise-ratio (SNR) as a parameter of image quality. Hence, the impact of the duration of ambient light exposure (which has a direct clinical impact) on important image quality parameters, such as brightness, contrast, contrast-to-noise ratio (CNR), SNR and image saturation of radiographs obtained with contemporary digital radiographic systems at different X-rays exposure times, remains unanswered.

There are multiple clinical conditions in the routine of dental practitioners in which PSP plates can be unintentionally exposed to light for varying durations, including those during which the PSP plate is partially unsheathed [7] or during a full-mouth series, power failure, and software issues, when the PSP plate is in a slightly loose opaque envelope [13]. Thus, the aim of this study is to quantitatively evaluate the influence of the duration of ambient light exposure on the image quality of digital radiographs obtained from contemporary PSP-based systems under different X-ray exposure times.

## 2. Materials and Methods

### 2.1. X-ray Exposure

**Figure 1:**
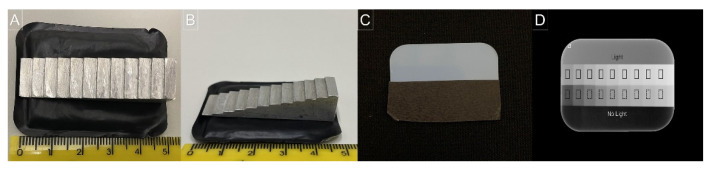
Setup for X-ray and light exposures. (**A**,**B**). Upper and lateral view of the aluminum step-wedge longitudinally fixed in the center of a sheathed size-2 PSP plate for X-ray exposure. (**C**). Upper view of the PSP plate with half of the sensitive surface covered with black paperboard for ambient light exposure. (**D**). Representative radiograph with the 18 ROIs: 9 in the exposed-to-light half (full-line rectangles) and 9 in the non-exposed-to-light half (dotted-line rectangles).

Ten repeated digital radiographs were obtained from an aluminum step-wedge composed of nine steps with an incremental thickness of 1 mm, longitudinally fixed in the center of a sheathed size 2 PSP plate (Figure 1A,B) of two digital radiographic systems: VistaScan Perio Plus (Durr Dental, Beitigheim-Bissingen, Germany) and Express (Instrumentarium Imaging, Tuusula, Finland). The X-ray source used for all exposures was the Focus unit (Instrumentarium, Tuusula, Finland) adjusted at 70 kV, 7 mA, a focus-to-image receptor distance of 30 cm, and five exposure times: 0.10, 0.20, 0.32, 0.40, and 0.50 s. The resulting dose–area product of each X-ray exposure time was, respectively, 21.93, 43.87, 70.19, 87.75, and 109.6 mGycm^2^.

### 2.2. Light Exposure and Scanning

After X-ray exposure but before scanning, half of the sensitive surface of the PSP plates was covered with black paperboard and the other half was exposed to ambient light (Figure 1C) for five durations: 5, 10, 30, 60, and 90 s. The duration of ambient light exposure was established based on certain clinical situations that routinely occur in dental schools and offices; the shorter durations (0–10 s) simulated when the opaque protection envelope is slightly loose or when the PSP scanner is designed in such a way that the plate is partially exposed to the ambient light when placed in the aperture of the transport slot of the scanner and the longer durations (30–90 s) simulated the time elapsed in a full-mouth series when multiple PSP plates are used.

Ambient light exposure and scanning occurred in a light-proof environment to avoid possible interference from uncontrolled sources of light. The ambient light was standardized to simulate a common clinical environment where the PSP scanner is located in a bright room by using a fluorescent lamp of 1380 lm, 23 W, 110 V, located 2.5 m away from the PSP plate. The resulting illuminance of this lamp on the surface of the PSP plate was measured using the Sekonic L-358 photometer (Sekonic, North White Plains, NY, USA) adjusted to the ambient-light-measurement mode, with the lumisphere extended and an ISO of 100, which registered 80 lux. Furthermore, for control purposes, a group of 10 PSP plates of each digital radiographic system was not exposed to ambient light.

**Figure 2:**
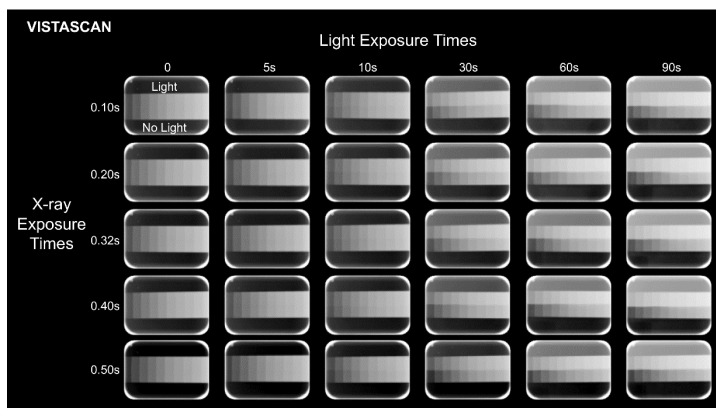
Representative radiographs of each experimental condition obtained using VistaScan. All images are shown using the same display window and level.

**Figure 3:**
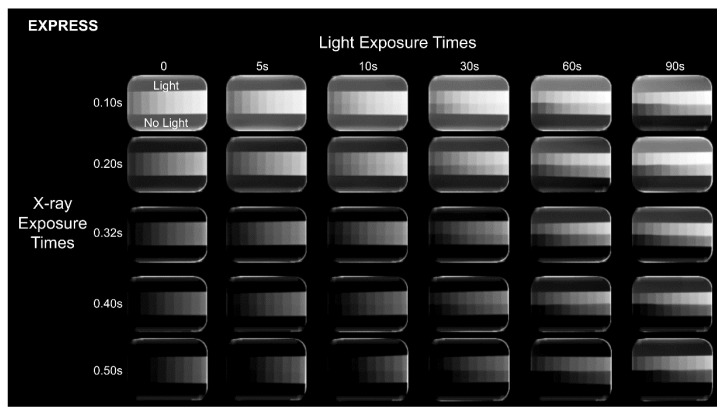
Representative radiographs of each experimental condition obtained using Express. All images are shown using the same display window and level.

All PSP plates were scanned immediately after ambient light exposure and the 600 resulting radiographs [10 repetitions × 2 digital radiographic systems × 5 X-ray exposure times × (5 ambient light exposure times + no light exposure)] were exported in the original file format (RAW). Representative radiographs of each experimental condition can be observed in Figure 2 and Figure 3. Also, because the VistaScan Perio Plus system is capable of simultaneously scanning 4 PSP plates, the PSP plates were placed in the same position and orientation in the cassette and scanned in the same transport slot to ensure highly controlled research conditions.

### 2.3. Image Evaluation

Using ImageJ software (National Institutes of Health, Bethesda, MD, USA), two regions of interest (ROI) were selected in nine fully exhibited steps of the aluminum step-wedge in all radiographs, such that one ROI was selected in the exposed-to-light half and the other one in the non-exposed-to-light half, totaling 18 ROIs per radiograph, as shown in Figure 1D. From each ROI, mean gray values (MGVs) and standard deviation of gray values were obtained.

Five metrics were assessed: brightness, contrast, CNR, SNR, and image saturation. Brightness was defined as the average of MGVs from the nine ROIs. Contrast was defined as the average of the differences of MGVs between adjacent pairs of aluminum steps. CNR was calculated for every two adjacent aluminum steps from both halves of the PSP plate and averaged. To calculate CNR, the difference of MGV between adjacent pairs of aluminum steps (represented by letters a and b in the following formula) was individually divided by the average of the standard deviation values of the same steps, as follows: $\mathrm{CNR}=\frac{{\mathrm{MGV}}_{b}-{\mathrm{MGV}}_{a}}{({\mathrm{SD}}_{a}+{\mathrm{SD}}_{b})/2}$. To obtain SNR, the MGV of each ROI was individually divided by the standard deviation and averaged. Finally, to express image saturation, the number of saturated steps—bright saturation (MGV ≥ 254) or dark saturation (MGV ≤ 1)—was counted in both halves of the PSP plate.

Then, the resulting values in the exposed-to-light half and the non-exposed-to-light half of each metric were individually subtracted and the resulting absolute values were averaged among the ten repeated radiographs. The final values will be referred to as discrepancy values.

### 2.4. Statistical Analysis

Exploratory data analysis was performed and transformations according to Box-Cox [14] were necessary (λ = 0.5 for brightness in both systems, λ = 0.5 for contrast in the VistaScan system, and λ = 0.35 for contrast in the Express system). After data transformation and using SAS software (SAS Software, Cary, NC, USA), version 9.4, analysis of variance (ANOVA) was applied whilst considering the effects of ambient light exposure time, X-ray exposure time, and the interaction between them. Multiple comparisons were performed using the Tukey test. The significance level was set at 5% (α = 0.05).

## 3. Results

**Figure 4:**
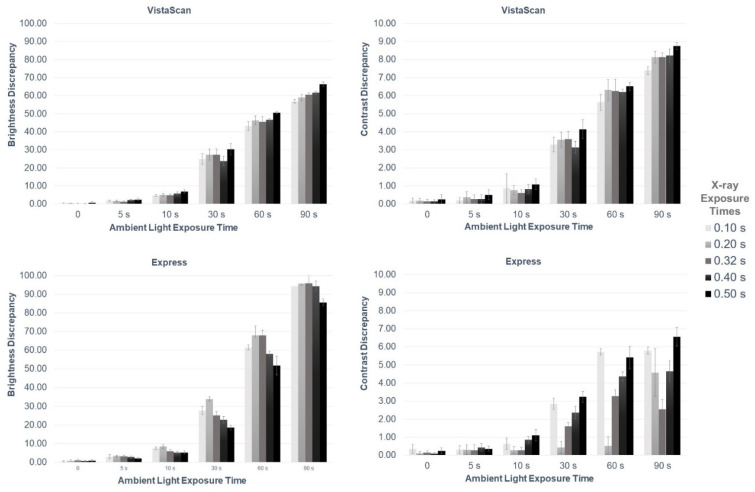
Bar graphs showing mean values of brightness and contrast discrepancies (in gray values) and standard deviations (error bars) as a function of exposure times of X-rays and ambient light for both digital radiographic systems.

As shown in Figure 4 and Table 1 and Table 2 the exposure of PSP plates to ambient light affected image brightness irrespective of the X-ray exposure time and digital radiographic system, with significantly greater brightness discrepancy at longer ambient light exposure times (*p* ≤ 0.05). MGVs in the exposed-to-light half of the PSP plates were higher than those in the non-exposed-to-light half. In most cases in VistaScan, longer X-ray exposure times led to a higher brightness discrepancy. In Express, the X-ray exposure time of 0.50 s presented the smallest brightness discrepancy at all ambient light exposure times (*p* ≤ 0.05).

The exposure of PSP plates to ambient light also affected image contrast irrespective of the X-ray exposure time and digital radiographic system, with significantly greater contrast discrepancy as of 30 s in Express and 10 s in VistaScan (*p* ≤ 0.05). When comparing contrast discrepancy among X-ray exposure times, no consistent behavior was observed for both systems (Figure 4 and Table 3 and Table 4). Mean contrast values in the exposed-to-light half of the PSP plates were higher than those in the non-exposed-to-light half.

The discrepancy in CNR and SNR values between the exposed- and non-exposed-to-light halves of the PSP plates increased when ambient light exposure time increased, irrespective of the digital radiographic system. The CNR and SNR values of the exposed-to-light half of the PSP plates were lower than that of the non-exposed-to-light half. Overall, for the same ambient light exposure, higher discrepancies in CNR values were observed at the lowest X-ray exposure times for both digital radiographic systems. As for SNR, at the lowest X-ray exposure times, higher discrepancies were observed for Express and lower discrepancies were observed for VistaScan.

At the X-ray and ambient light exposure times used in this study, no bright saturation (MGV ≥ 254) was observed in either digital system; dark saturation (MGV ≤ 1) was only identified in the Express system at X-ray exposure times of 0.40 and 0.50 s. Interestingly, the number of dark-saturated steps was greater in the non-exposed than in the exposed-to-light half of the PSP plates (2 steps versus 1 step, respectively). The dark saturation discrepancy between the exposed- and non-exposed-to-light halves of the PSP plates was directly proportional to the ambient light exposure times, except for at 90 s.

The discrepancy values observed in Figure 4 and Table 1, Table 2, Table 3 and Table 4 increased most notably as of 10 s of light exposure, irrespective of the X-ray exposure time and digital radiographic system.

## 4. Discussion

The duration of ambient light exposure is directly proportional to its negative effect on the image quality of PSP-based radiographs. Overall, increased ambient light exposure time led to significantly higher discrepancies in brightness, contrast, SNR, and CNR, irrespective of the X-ray exposure time and digital radiographic system. Furthermore, only in Express was the dark saturation observed at the longest X-ray exposure times—0.40 and 0.50 s—reduced at 90 s of ambient light exposure. All ambient light exposure times evaluated—from 5 s to 90 s—affected brightness, contrast, and CNR. Therefore, based on this in vitro setup, we would recommend avoiding any ambient light exposure of PSP plates in the time that elapses between exposure to X-rays and scanning. However, although follow-up clinical studies are needed to assess the effect of ambient light on different diagnostic tasks, when we consider our outcomes, it seems reasonable to suggest 10 s as the maximum ambient light exposure time (Figure 4 and Table 1, Table 2, Table 3 and Table 4), since both brightness and contrast discrepancy values were more prominent as of 30 s.

Discrepancy values are considered to be a good strategy to assess the effect of ambient light on PSP plates and more appropriate for comparison purposes than the absolute digital values because only the former can reflect good versus bad behavior, which means that greater discrepancy is harmful and reduced discrepancy is beneficial. When it comes to the absolute MGV and contrast values, greater and lower values do not necessarily correlate with being good or bad and strongly depend on clinical validation.

Radiographic image quality can be assessed through numerous metrics such as brightness, contrast, SNR, and CNR; an ideal radiographic image should reveal intermediate levels of these parameters. Brightness reveals the intensity of a pixel, contrast represents the difference between pixel intensities [15], CNR is the balance between contrast and noise [15,16], and SNR is related to the image quality of a digital radiograph [2]. Accordingly, higher CNR values represent a satisfactory balance: when image contrast outweighs image noise. In the present study, CNR and SNR decreased when ambient light exposure time increased, which is in agreement with previous studies [2,7].

Although previous studies have addressed the effect of delayed scanning on the image quality of PSP plates [9,11,12,17,18], none of them isolated the impact of ambient light exposure on the image quality of PSP plate-based radiographs. We strongly believe that the increased image brightness and contrast observed after light exposure within the durations proposed in the present study might be a consequence of the directly proportional erasing of PSP plates. Because the clinical relevance of brightness and contrast adjustments for image quality is user-specific, it is important to highlight that the present methodological design focused only on detection of the discrepancy caused by ambient light exposure; pre-clinical and clinical studies are needed to further assess the effect on multiple diagnostic tasks.

The present study also evaluated the effect of ambient light on image saturation, which is when the limits of the grayscale are reached. No cases of bright saturation (MGV ≥ 254) were observed in the present study, only dark saturation (MGV ≤ 1). Although PSP plate-based digital radiographic systems present a longer dynamic range than analog films and solid-state sensors [1,16], the selection of X-ray exposure time must be accurate to avoid unnecessary patient exposure [16]. Interestingly, in the present study, the exposure of PSP plates to light for 90 s reduced dark saturation. Intriguingly, this may reveal that ambient light exposure was beneficial for that specific condition. Despite this, assuming that ambient light exposure erases PSP plates, such ambient light exposure may have partially erased the dark steps and increased the MGVs. It is unlikely that this effect translates to clinical images, i.e., it will not allow one to recover anatomical details that are lost due to overexposure. Furthermore, based on radiation protection principles, X-ray overexposure cannot be ignored and compensated for by means of ambient light exposure.

For the same ambient light exposure, X-ray exposure times led to some slightly different behaviors in the effect of ambient light on image brightness and contrast between the two digital radiographic systems used in this study. Although both digital radio-graphic systems make use of PSP plates, we believe that such differences could be related to their dynamic ranges. A previous study [16] assessed radiographic images of human mandibles and revealed that VistaScan has a greater dynamic range than Express, which could be the reason why contrast discrepancy was, in general, more impactful in VistaScan. As for some non-uniform results in Express, we believe that some technical specifications not disclosed by the manufacturers—for instance, the exact composition of the PSP plate, actual sensitivity, scanning laser beam width, and scan direction—may be the cause of such differences; no possible effect from digital postprocessing was considered because we intentionally only made use of RAW files. It is important to consider the significant role of manufacturers in further developing currently marketed PSP-based systems by possibly increasing the energy-sensitivity of the materials to respond solely or primarily to X-rays rather than to visible light. However, we recognize that the materials and methods could be quite costly and thus not marketable for the time being. Importantly, our study focused on contemporary PSP-based systems, which can be expected to have the most advanced technology.

The image quality of PSP plate-based radiographs was quantitatively evaluated in this study and found to be affected by ambient light exposure. Considering the in vitro nature of the present study, our methodological approach allowed us to have absolute control over external factors to isolate the impact of ambient light and X-ray exposures on the image quality of PSP plate-based radiographs. Further studies qualitatively assessing the impact of ambient light on PSP plates on different diagnostic tasks are encouraged.

## 5. Conclusions

The negative effects of ambient light exposure on the image quality of PSP-based radiographs are directly proportional to the duration of exposure. The effects include increased brightness and contrast and decreased CNR, SNR, and dark saturation. Clinicians should be aware of such harmful effects when handling and scanning PSP plates in bright environments.

## Figures and Tables

**Table 1:** Mean values, standard deviation (SD), and pairwise significance (Sig.) of brightness discrepancy as a function of light and X-ray exposure times for the VistaScan system.

X-ray Exposure Time (s)	Light Exposure Time (s)																	
	0			5			10			30			60			90		
	Mean	SD	Sig.	Mean	SD	Sig.	Mean	SD	Sig.	Mean	SD	Sig.	Mean	SD	Sig.	Mean	SD	Sig.
**0.1**	0.38	0.22	Aab	1.82	0.31	Bab	4.75	0.64	Cb	24.89	2.86	Db	43.22	2.39	Eb	56.84	0.93	Fb
**0.2**	0.31	0.20	Aab	1.68	0.41	Bab	5.08	0.84	Cb	27.33	3.14	Da	46.37	2.39	Eab	58.97	1.72	Fb
**0.32**	0.25	0.13	Aab	1.37	0.46	Bb	4.94	0.57	Cb	27.42	2.99	Da	45.56	2.85	Eb	60.48	1.16	Fb
**0.4**	0.16	0.13	Ab	2.31	0.43	Ba	5.76	0.81	Cab	23.83	2.60	Db	46.59	0.76	Eab	61.60	0.90	Fab
**0.5**	0.65	0.50	Aa	2.50	0.70	Ba	6.95	0.92	Ca	30.44	3.08	Da	50.67	0.69	Ea	66.39	1.16	Fa

*p*-values from ANOVA representing overall effects: *p* (Exposure time) < 0.0001; *p* (Light Condition) < 0.0001; *p* (Exposure time × Light Condition) < 0.0001. Pairwise comparisons from Tukey’s test are shown under the significance (Sig.) columns: mean values followed by distinct uppercase letters differ significantly between ambient light exposure times for the same X-ray exposure time (i.e., comparisons within the same row) and mean values followed by distinct lowercase letters differ significantly between X-ray exposure times for the same ambient light exposure time (i.e., comparisons within the same column).

**Table 2:** Mean values, standard deviation (SD), and pairwise significance (Sig.) of brightness discrepancy as a function of light and X-ray exposure times for the Express system.

X-ray Exposure Time (s)	Light Exposure Time (s)																	
	0			5			10			30			60			90		
	Mean	SD	Sig.	Mean	SD	Sig.	Mean	SD	Sig.	Mean	SD	Sig.	Mean	SD	Sig.	Mean	SD	Sig.
**0.1**	0.49	0.29	Aa	3.03	0.31	Bab	7.47	0.72	Cab	27.75	2.17	Db	61.34	1.35	Eb	94.18	1.18	Fa
**0.2**	0.66	0.64	Aa	3.31	0.41	Ba	8.51	0.82	Ca	33.80	1.23	Da	68.02	5.00	Ea	95.70	2.35	Fa
**0.32**	0.95	0.73	Aa	3.17	0.46	Bab	5.95	0.87	Cb	25.19	2.00	Dc	68.01	2.81	Ea	95.87	6.26	Fa
**0.4**	0.65	0.31	Aa	2.76	0.43	Bab	5.16	0.59	Cc	22.80	1.77	Dc	57.96	1.42	Eb	94.30	2.74	Fa
**0.5**	0.88	0.41	Aa	2.10	0.70	Bb	5.24	0.84	Cc	18.55	1.22	Dd	51.82	4.97	Ec	85.60	1.81	Fb

*p*-values from ANOVA representing overall effects: *p* (Exposure time) < 0.0001; *p* (Light Condition) < 0.0001; *p* (Exposure time × Light Condition) < 0.0001. Pairwise comparisons from Tukey’s test are shown under the significance (Sig.) columns: mean values followed by distinct uppercase letters differ significantly between ambient light exposure times for the same X-ray exposure time (i.e., comparisons within the same row) and mean values followed by distinct lowercase letters differ significantly between X-ray exposure times for the same ambient light exposure time (i.e., comparisons within the same column).

**Table 3:** Mean values, standard deviation (SD), and pairwise significance (Sig.) of contrast discrepancy as a function of light and X-ray exposure times for the VistaScan system.

X-ray Exposure Time (s)	Light Exposure Time (s)																	
	0			5			10			30			60			90		
	Mean	SD	Sig.	Mean	SD	Sig.	Mean	SD	Sig.	Mean	SD	Sig.	Mean	SD	Sig.	Mean	SD	Sig.
**0.1**	0.19	0.13	Aa	0.20	0.15	Aa	0.87	0.81	Bab	3.29	0.41	Cb	5.63	0.45	Da	7.39	0.20	Eb
**0.2**	0.18	0.12	Aa	0.36	0.31	Aa	0.76	0.26	Bab	3.56	0.42	Cab	6.32	0.57	Da	8.12	0.33	Eab
**0.32**	0.15	0.12	Aa	0.27	0.23	Aa	0.62	0.18	Bb	3.60	0.42	Cab	6.26	0.64	Da	8.13	0.23	Eab
**0.4**	0.14	0.09	Aa	0.27	0.23	Aa	0.83	0.25	Bab	3.13	0.35	Cb	6.20	0.15	Da	8.22	0.37	Eab
**0.5**	0.25	0.26	Aa	0.51	0.28	Aa	1.09	0.29	Ba	4.14	0.52	Ca	6.51	0.22	Da	8.75	0.20	Ea

*p*-values from ANOVA representing overall effects: *p* (Exposure time) < 0.0001; *p* (Light Condition) < 0.0001; *p* (Exposure time × Light Condition) = 0.0432. Pairwise comparisons from Tukey’s test are shown under the significance (Sig.) columns: mean values followed by distinct uppercase letters differ significantly between ambient light exposure times for the same X-ray exposure time (i.e., comparisons within the same row) and mean values followed by distinct lowercase letters differ significantly between X-ray exposure times for the same ambient light exposure time (i.e., comparisons within the same column).

**Table 4:** Mean values, standard deviation (SD), and pairwise significance (Sig.) of contrast discrepancy as a function of light and X-ray exposure times for the Express system.

X-ray Exposure Time (s)	Light Exposure Time (s)																	
	0			5			10			30			60			90		
	Mean	SD	Sig.	Mean	SD	Sig.	Mean	SD	Sig.	Mean	SD	Sig.	Mean	SD	Sig.	Mean	SD	Sig.
**0.1**	0.35	0.24	Ba	0.32	0.22	Ba	0.64	0.31	Ba	2.84	0.31	Ca	5.72	0.18	Da	5.79	0.20	Dab
**0.2**	0.11	0.09	Bab	0.30	0.27	CBa	0.28	0.19	CBb	0.42	0.33	Cc	0.53	0.49	Cb	4.57	1.34	Db
**0.32**	0.15	0.11	Bab	0.29	0.29	Ba	0.27	0.17	Bb	1.60	0.21	Cb	3.28	0.36	Db	2.55	0.56	DCc
**0.4**	0.09	0.07	Ab	0.43	0.21	Ba	0.86	0.19	Ba	2.36	0.34	Cab	4.37	0.25	Dab	4.64	0.57	Dab
**0.5**	0.25	0.15	Aab	0.36	0.15	Aa	1.11	0.32	Ba	3.24	0.31	Ca	5.41	0.62	Da	6.57	0.52	Da

*p*-values from ANOVA representing overall effects: *p* (Exposure time) < 0.0001; *p* (Light Condition) < 0.0001; *p* (Exposure time × Light Condition) < 0.0001. Pairwise comparisons from Tukey’s test are shown under the significance (Sig.) columns: mean values followed by distinct uppercase letters differ significantly between ambient light exposure times for the same X-ray exposure time (i.e., comparisons within the same row) and mean values followed by distinct lowercase letters differ significantly between X-ray exposure times for the same ambient light exposure time (i.e., comparisons within the same column).
